# Customized MFM probes with high lateral resolution

**DOI:** 10.3762/bjnano.7.100

**Published:** 2016-07-25

**Authors:** Óscar Iglesias-Freire, Miriam Jaafar, Eider Berganza, Agustina Asenjo

**Affiliations:** 1 Instituto de Ciencia de Materiales de Madrid (ICMM-CSIC), Calle Sor Juana Inés de la Cruz 3, 28049, Madrid, Spain; 2 Department of Physics, McGill University, 3600 rue University, H3A 2T8, Montreal, Canada

**Keywords:** atomic force microscopy (AFM), AFM probes, high-resolution microscopy, magnetic force microscopy (MFM), magnetic materials

## Abstract

Magnetic force microscopy (MFM) is a widely used technique for magnetic imaging. Besides its advantages such as the high spatial resolution and the easy use in the characterization of relevant applied materials, the main handicaps of the technique are the lack of control over the tip stray field and poor lateral resolution when working under standard conditions. In this work, we present a convenient route to prepare high-performance MFM probes with sub-10 nm (sub-25 nm) topographic (magnetic) lateral resolution by following an easy and quick low-cost approach. This allows one to not only customize the tip stray field, avoiding tip-induced changes in the sample magnetization, but also to optimize MFM imaging in vacuum (or liquid media) by choosing tips mounted on hard (or soft) cantilevers, a technology that is currently not available on the market.

## Introduction

Conventional MFM probes consist of pyramidal Si or SiN tips with a ferromagnetic thin film coating (generally a CoCr alloy) mounted on a cantilever with resonance frequency and spring constant of around 75 kHz and 3 N/m, respectively, and with a final apex radius of typically tens of nanometres. Plenty of literature can be found that reports on probe engineering aiming to achieve enhanced spatial resolution. Several works focus on reducing the physical size of the magnetic material of the tip, either by using focused ion beam (FIB) milled tips [1–2], electron beam deposited tips [3–4] or stencil-deposited metal dots onto an AFM tip [5]. Following a different approach, probes with carbon nanotubes (CNTs) have been fabricated for MFM imaging either by mechanical attachment [6–8] or direct growth on commercial pyramid tips [9]. Although good control in terms of angle and position can be achieved when attaching CNTs to Si tips by using nanomanipulators [10], it requires sophisticated and time-consuming processes. Other approaches use magnetic nanowires [11] or coated carbon nanocones [12] to improve the detection of small domains. One can also find the use of nanomagnets with high anisotropy as MFM probes [13] and different approaches to control the final domain at the tip apex [14–15], seeking best sensitivity or resolution. However, the easiest interpretation of the results is possible in the case of single-domain MFM tips with negligible influence over the domain structure of the sample [16].

In general, the aforementioned methods involve a considerable amount of time, effort and the use of advanced fabrication techniques for engineering the tips. In addition, the achievable resolution is not greatly enhanced compared to commercial tips so their applicability is limited to particular cases of interest. Intrinsically related to the lateral resolution is the magnetic sensitivity of the probe. In order to achieve better signal-to-noise ratios, one may want larger amounts of magnetic material to be deposited at the tip apex. Unfortunately, this results in larger tip radii and subsequent lower lateral resolution; furthermore, the influence over the sample magnetic state can increase. Depending on the specific properties of each sample, a balance between resolution and sensitivity must be found. For this reason, different kinds of commercial MFM probes are available for sale. Specific low-moment or side-coated probes (having lower amounts of ferromagnetic material) can be found in the market. However, best lateral resolution is achieved with super-sharp tips. All these specific probes are found in the market at higher prices than the standard ones.

The importance of choosing a proper tip becomes clear when measuring soft samples [17–18]. Often even the stray field emerging from low-moment commercial probes becomes too large for that kind of samples. Our goal herewith is to go a step further and be able to customize the tip used for each case, particularly regarding the irreversible influence the stray field might have in magnetically soft samples. Therefore, this work is not focused on pushing the limits of MFM resolution; instead, we aim to fabricate customized tips with high lateral resolution and a controlled influence in the sample by using a fast and rather accessible approach. Our method is based on easily preparing MFM probes from commercial AFM probes by using a specific coating. By doing so, we can tune the amount of magnetic material in the probe and also select the cantilever properties for each experiment, such as the spring constant, resonance frequency or the position of the tip on the lever. We have found neither in the literature nor in the market any MFM probe with cantilever characteristics far from the range of the aforementioned. Making MFM accessible to these properties in any lab is particularly useful if one wants to carry out experiments in environments such as vacuum or liquid [19].

## Experimental

The custom-made tips presented in this manuscript are fabricated by sputter coating commercial AFM sensors with a polycrystalline Co thin film with no buffer layer, using a custom-built AC magnetron sputtering system with a substrate holder designed on purpose to favour the growth of the magnetic layer on one side of the pyramidal tip. The deposition parameters are carefully chosen to yield highly flat surfaces with small grain size, ensuring higher lateral resolution for the MFM tips [20] (more details on the deposition parameters can be found in section 1 of Supporting Information File 1).

Even though sputtering is not a highly directional technique, there is a preferential direction of deposition. Thus, by selecting a specific incidence angle, it is possible to cover mainly one side of the tip pyramid. A residual magnetic film with sub-nanometre thickness was found on the remaining parts of the tip–cantilever system. No capping layer is subsequently deposited onto the Co film since, although the outermost atomic layers of cobalt are well known to oxidize under ambient conditions, this affects a very superficial region of the coating of around 2 nm [21]. This oxide layer does not avoid obtaining good MFM imaging results even several months after deposition, with no measured loss of sensitivity and one then avoids the undesired increase in the tip radius caused by the capping material. Magnetic layers of different thickness can be deposited depending on the characteristics of the sample under study. For clarity, we focus here on tips yielding an optimal balance between sensitivity and lateral resolution that can be used with a broad range of samples, having a nominal Co layer of either 20 or 25 nm. Single crystalline silicon substrates were placed close to the AFM tips during deposition and used as reference samples for vibrating sample magnetometry (VSM) and AFM/MFM characterization. In all the experiments shown hereafter MFM data correspond to the shift in the resonance frequency of the cantilever recorded during the retrace scan (withdrawing the sample by 10–20 nm from the topographic set point distance) by using a phase locked-loop (PLL) feedback.

**Figure 1:**
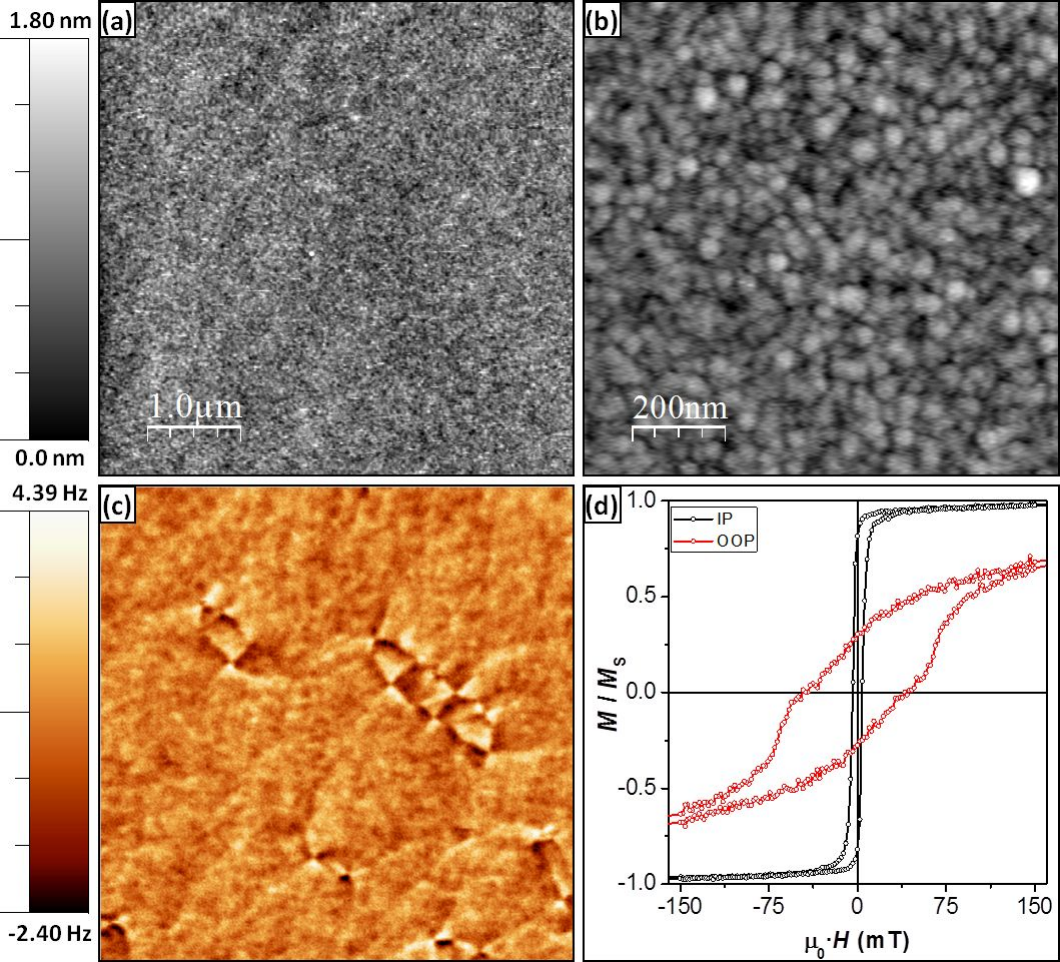
(a) (5 × 5) μm^2^ and (b) (750 × 750) nm^2^ topographic images of a 20 nm thick Co layer grown onto a silicon substrate under the conditions used for coating the tips. (c) (5 × 5) μm^2^ MFM image of the same sample, showing a predominant IP magnetization with the presence of cross-tie domain walls. (d) VSM hysteresis loops show the preference for the magnetization to remain IP. Note: (a) & (c) were recorded simultaneously using a custom-made tip with a 20 nm Co coating on one side, whereas (b) was measured using a commercial AFM probe.

The topography and the magnetic properties of the reference Co/Si sample are shown in Figure 1a–c. On the one hand, a resulting flat surface with a root mean square (RMS) roughness of (0.25 ± 0.05) nm is obtained. The mean grain size, extracted through the self-correlation function along the fast scan direction in Figure 1b, is around 20 nm (notice that such value is an overestimation due to the tip–sample convolution effect). Both parameters were not found to change in great measure for the thickness range used to coat the AFM sensors. On the other hand, the MFM analysis demonstrates that the easy axis of this thin film remains mainly in-plane (IP) due to the influence of the dominating shape anisotropy (as deduced from the cross-tie domain walls [22] seen in Figure 1c), in agreement with the macroscopic hysteresis loops measured at room temperature by VSM (Figure 1d). A high remanent magnetization of 82% of the saturation value is found when an IP field is applied. This, together with the shape anisotropy induced by the pyramidal tip, forces the magnetic moments to remain mainly oriented along the pyramid surface. Nevertheless, the orientation of the spins at the apex will depend on the apex shape and on the way the cobalt layer covers it.

## Results and Discussion

### Magnetization reversal process of the custom-made probes

A non-conventional MFM-based method previously reported [23] was used to measure local hysteresis loops of the MFM probes [24–25] (see section 2 of Supporting Information File 1 for more details). This approach allows for measuring the magnetostatic tip–sample interaction as a function of the magnetic field. By assuming that the sample magnetization remains unchanged during the experiment (*H*_c_ >> *H*_applied_), as is commonly assumed to be the case for magnetic hard disks, one can gain insight into the evolution of the spins at the tip apex with the external field and extract the intrinsic hysteresis loop of the MFM tip [25]. Typically, a large Barkhausen jump is observed with a well-defined switching field. By doing so, the measured average switching field for the 20 nm Co homemade tip is μ_0_∙<*H*_switch_> = (31 ± 4) mT, where a total of 30 experiments was performed including three different custom-made probes. This value is within the range of those obtained for commercial probes [24] and allows for reliable MFM measurements in a wide range of applied fields.

Additionally, the tip radius of the homemade probes was evaluated before and after deposition of the magnetic coating by imaging of a reference sample with carbon nanotubes. Refer to section 3 of Supporting Information File 1 for further detail.

### Sensitivity and lateral resolution of the custom-made probes

In order to evaluate the lateral resolution of the probes coated on one side, we present a comparison of the MFM images obtained with four different sorts of probes: three kinds of commercial tips [26–27] and the custom-made probes described here, all of them using cantilevers of similar properties (namely resonance frequency and spring constant of around 75 kHz and 3 N/m, respectively). For these experiments, a high density hard disk with perpendicular anisotropy was used, based on a CoCrPt alloy and courtesy of Toshiba. The domain size is approximately 25 nm.

**Figure 2:**
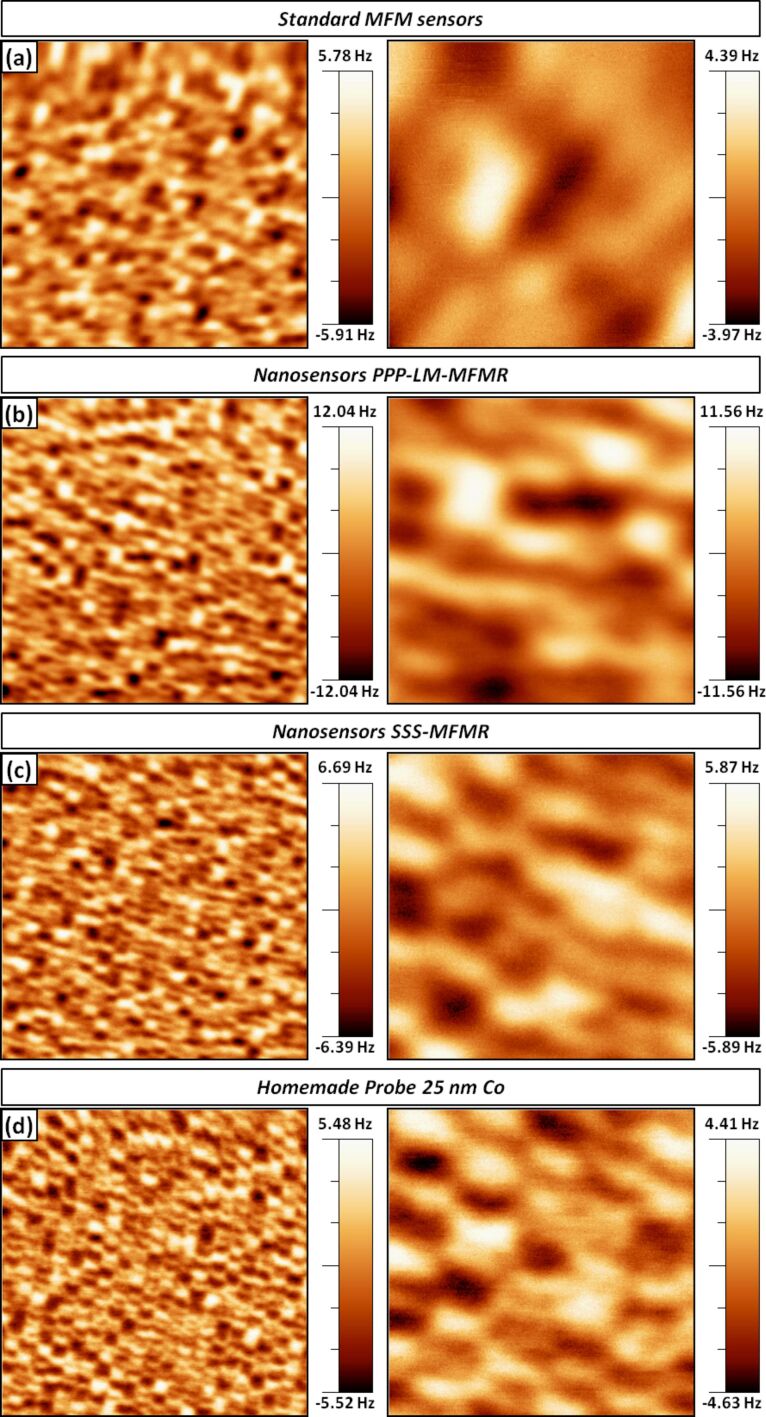
MFM images performed to show the lateral resolution obtained with commercial (a) standard, (b) low-moment and (c) super sharp probes and (d) custom-made tips with a 25 nm thick Co coating on the front sides. Images on the left/right columns are (2 × 2) μm^2^/(500 × 500) nm^2^, respectively.

Three pristine probes of each type were chosen and standard MFM images were measured with analogous parameters (oscillation amplitude ≈ 14 nm, retrace distance from the topographic set point ≈ 15 nm); the best results obtained for each set are shown in Figure 2. The first kind of probes is a standard model with a nominal resolution of about 50 nm, thus being suitable to characterize domains hundreds of nanometers in size or for testing experiments. The second type (low moment probes) is meant for relatively soft samples and has a nominal resolution of 35 nm, showing an improved performance as compared to the previous ones. The third option is the so-called super-sharp MFM tips, which are specific for performing high resolution magnetic force microscopy measurements (with a nominal resolution of 25 nm). These tips with high aspect ratio are particularly suitable for single-pass non-contact MFM [28], a mode that becomes particularly useful when measuring soft magnetic samples with very flat surfaces under ultra-high-vacuum (UHV) conditions, as it prevents the tip from tapping the sample surface and helps preserving its sharpness.

In the last row, data from a custom-made probe with a one-sided Co coating of 25 nm are shown. Having a look at the column on the left, where (2 × 2) μm^2^ images are displayed, an intrinsic lower resolution of the first two types of commercial probes is readily deduced. On the contrary, the super-sharp and the custom-made tips seem to have comparable performances. The difference to the two first kinds of tips becomes even more evident when the image size is decreased down to (500 × 500) nm^2^, as seen on the right column. In the first case, only large domains –composed of large series of neighbouring domains with parallel magnetization– are resolved (Figure 2a). Using low-moment MFM tips, quite better results are obtained (Figure 2b) but the lateral resolution is far from being enough to resolve individual domains. In agreement with its label name, the super-sharp tips yield the best resolved images of all commercial probes (Figure 2c). Nevertheless, at least as good results are obtained with the custom-made sensors, as observed in Figure 2d.

**Figure 3:**
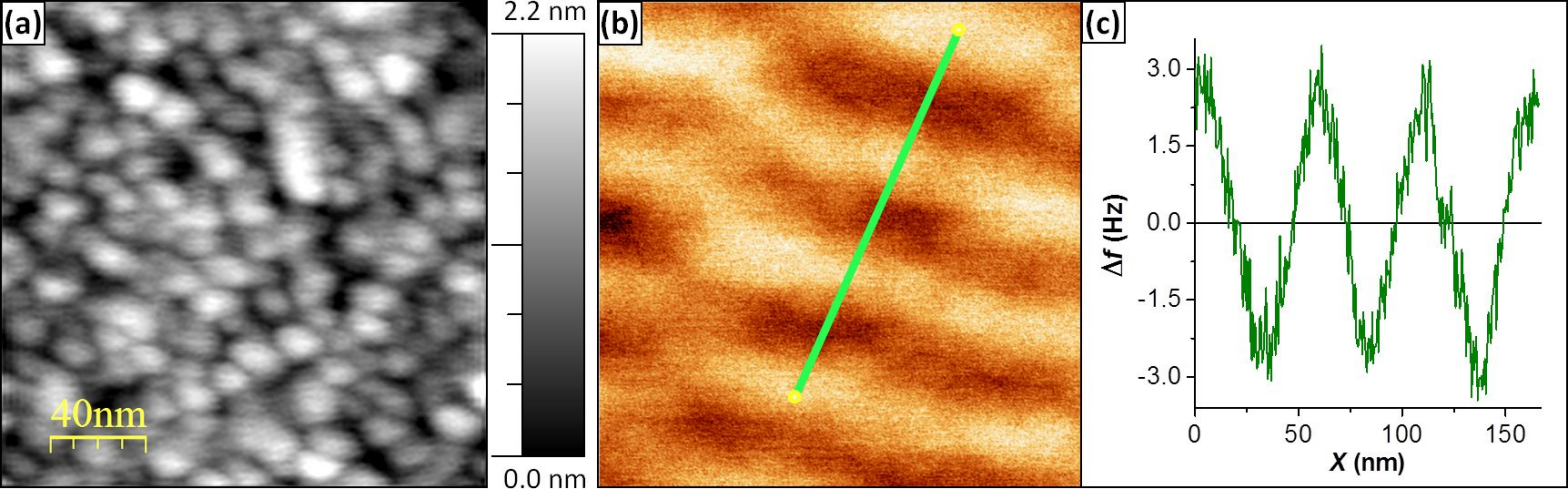
(a) Topography of a high-density HDD, recorded with a custom-made tip with 20 nm coating. The lateral resolution is below 10 nm. (b) Corresponding MFM image showing single domains with alternating OOP orientations. (c) Frequency shift profile along the line shown in (b) that gives an inter-domain distance of about 25 nm.

Depending on the sample one might need to even further increase the lateral resolution. This could be achieved by reducing the thickness deposited onto the AFM tips at the expense of reducing their magnetic sensitivity. Figure 3 displays the topography and MFM image of a different region of the same hard disk obtained by a custom-made tip with a 20 nm thick coating. Figure 3a shows grains of the order of 10 nm in size, a remarkable resolution even for non-magnetic AFM probes. The corresponding MFM image shows series of bright and dark stains associated to domains with alternate OOP magnetization (Figure 3b). The frequency shift along the profile drawn is displayed in Figure 3c and confirms the inter-domain distance to be around 25 nm. Note that no smoothing has been applied to the profile data.

In addition to the standard cantilevers typically used to perform MFM imaging, probes with non-standard mechanical properties were prepared to give a glimpse of the potential applicability that can be explored with customized MFM probes. Examples of MFM tips mounted on hard (*k* ≈ 40 N/m) cantilevers, commonly used for imaging in vacuum, and soft (*k* ≈ 0.09 N/m) levers, used to map soft biological samples, are given in the section 4 of Supporting Information File 1. Please refer there for further information.

The smaller amount of magnetic material deposited onto the tips shown here make them intrinsically excellent candidates for MFM imaging of soft samples. By using the method described in [24] and [29] –in which the magnetostatic influence exerted on an array of single-domain nickel nanowires embedded in an Al_2_O_3_ membrane was used to calibrate the stray field of the MFM tip– we can estimate the value of the stray field emerging from the custom-coated tips and compare it to the stray field of commercial tips. The axial field estimated for a custom-coated tip with a 25 nm cobalt thickness is less than 25 mT. This value is smaller than any of those reported for different commercial models, making the tips reported here optimal for imaging of soft samples. Finally, similar stray fields than those measured for standard commercial probes can be obtained by depositing thicker films (ca. 70 nm). The in-plane (IP) component of the MFM probes can also be relevant depending on the domain structure, a good example of which is the vortex configuration. Again, lower IP field values are attributed to the custom-coated probes [18,20].

## Conclusion

In this work we describe an accessible route for customizing MFM probes to desire by coating one side of standard AFM tips using sputtering. We demonstrate that the resulting probes achieve lateral resolutions at least as good as commercial super-sharp tips (10 nm and 25 nm in topographic and MFM images, respectively). A particular advantage is the possibility of coating tips mounted on hard (*k* ≈ 40 N/m)/soft (*k* ≈ 0.09 N/m) cantilevers to optimize imaging in vacuum/liquid environments, providing enhanced sensitivity compared to standard probes.

## Supporting Information

File 1 Additional experimental data.
