# Supplementary material for: Customized MFM probes with high lateral resolution
Source: Beilstein J Nanotechnol. 2016 Jul 25;7:1068–74. doi: 10.3762/bjnano.7.100 (PMC4979883; doi:10.3762/bjnano.7.100)
Supplement: File 1 — Additional experimental data. [file Beilstein_J_Nanotechnol-07-1068-s001.pdf]

Supporting Information

for

Customized MFM probes with high lateral resolution

Óscar Iglesias-Freire*[1,2], Miriam Jaafar[1], Eider Berganza[1] and Agustina Asenjo[1]

Address: [1]Instituto de Ciencia de Materiales de Madrid (ICMM-CSIC), Calle Sor Juana Inés de la Cruz 3 (28049, Madrid) Spain and [2] Department of Physics, McGill University, 3600 rue University (H3A 2T8, Montreal) Canada

Email: Óscar Iglesias-Freire* - oiglesias@physics.mcgill.ca

* Corresponding author

Additional experimental data

1 Properties of the thin films as a function of the deposition parameters

Starting from a base pressure of $5 \cdot 10^{-6}$ mbar, argon gas is introduced into the chamber until the selected working pressure is reached. Inert gases are usually employed as the sputtering gas because they tend not to react with the target material and because of their high molecular weight that causes higher sputtering rates. Plasma is then generated using an *AC* magnetron sputtering with an input power of 100 W and is confined on the target surface by a permanent magnet located behind the target surface. Positively charged Ar^{+} ions are accelerated toward the negatively biased 99.99% pure Co target, resulting in material being sputtered and deposited onto the side walls of the tips.

Table S1 shows the influence of the working pressure in the properties of constant-thickness reference samples. As can be observed, the in-plane coercive field (measured by VSM) changes for different working pressures. This parameter is of importance when an in situ magnetic field is applied during the MFM operation.

Table S1: Influence of the working pressure over the reference sample.

deposition parameters		properties of the thin film	
pressure (mbar)	Co thicknesss (nm)	RMS (nm)	$\mu_0 \cdot H_C^{IP}$ (mT)
$2.5 \cdot 10^{-2}$	45	1.3	22
$1.9 \cdot 10^{-2}$	45	1.0	23
$1.0 \cdot 10^{-2}$	45	0.6	12
$8.6 \cdot 10^{-4}$	45	0.4	6.5

The reference samples were characterized by AFM and also by MFM. In every case, the average grain size is below 14 nm, whereas the magnetization of the grown films remains constant with a value of $8{\cdot}10^2$ emu/cm^3.

Figure S1 shows the evolution of the magnetic configuration as a function of the working pressure used during the deposition. The domain configuration evolves from dense stripes domains for the higher pressures to cross-tie domain walls for smaller pressures.

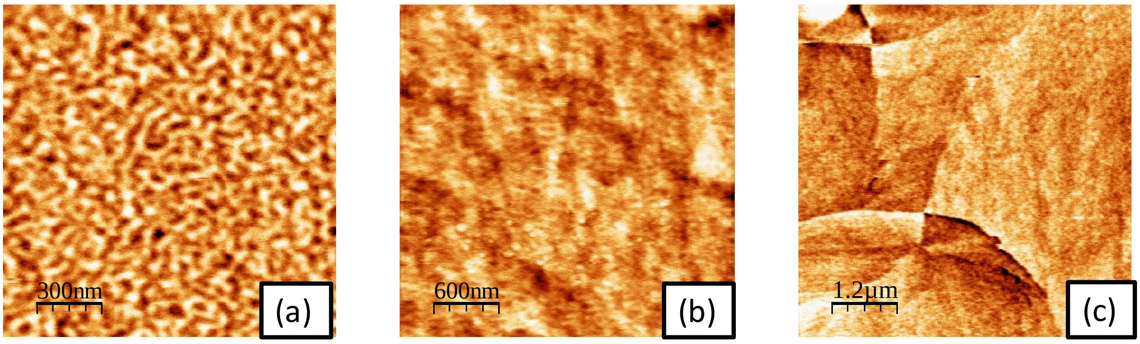

Figure S1: MFM images of 45 nm thick Co films, for different working pressures: (a) P = $2.5{\cdot}10^{-2}$ mbar. Oscillations of the out-of-plane component of the magnetization can be observed, (b) P = $1.0{\cdot}10^{-2}$ mbar. A dense stripe domain configuration is observed, (c) P = $8.6{\cdot}10^{-4}$ mbar. Cross-tie DWs are a fingerprint of the magnetization lying mainly in plane.

Data in Table S2 show the influence of the magnetic layer thickness on the magnetic behavior. The working pressure was kept around $1.0{\cdot}10^{-2}$ mbar (the same chosen for the experiments presented in the manuscript). The effect the thickness has on the magnetic properties of a thin film is well known. A critical thickness (t_c) was observed to obtain the so-called dense stripe domains [1], whose value is a function of the saturation magnetization and the perpendicular anisotropy constant, K_u.

Table S2: Influence of the thickness of the magnetic layer on the magnetic properties.

deposition parameters		properties of the thin film	
pressure (mbar)	Co thicknesss (nm)	RMS (nm)	$\mu_0 \cdot H_C{}^{IP}$ (mT)
$1.0 \cdot 10^{-2}$	15	0.5	4.5
$1.0 \cdot 10^{-2}$	30	0.6	9.3
$1.0 \cdot 10^{-2}$	45	0.6	12
$1.0 \cdot 10^{-2}$	60	0.6	27
$1.0 \cdot 10^{-2}$	75	0.7	27

MFM measurements allow us to follow the evolution of the domain configuration as a function of film thickness. The thinnest layers present mainly in-plane magnetization with the presence of cross-tie domain walls (see Figure S1c). As the Co layer thickness increases, the out-of-plane component of the magnetization increases until a dense stripe domain configuration is seen for the layer 60 nm thick (Figure S2c). These MFM results are in good agreement with hysteresis loops measured by VSM.

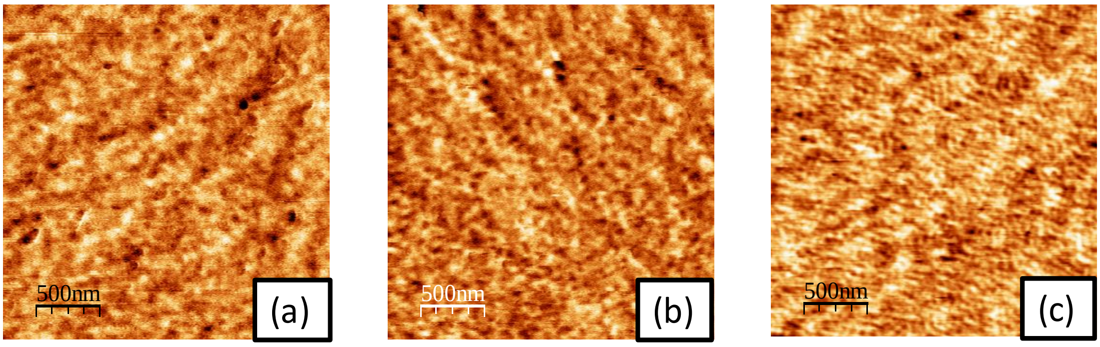

Figure S2: MFM images of Co thin films deposited under an Ar pressure of P = $1.0 \cdot 10^{-2}$ mbar. The layer thickness is (a) 30 nm, (b) 45 nm and (c) 60 nm. The thicker layer presents dense stripe domains.

2 Characterization of the switching field of the custom-made MFM probes

The resulting custom-coated MFM probes were characterized using an advanced MFM method described elsewhere [2]. As sketched in Figure S3a, a standard hard disk is mapped and conventional topographic and MFM images are obtained first (Figure S3b). Then, the slow scan direction is disabled; instead, an external out-of-plane (OOP) field is swept after each fast scan. The resulting $\Delta f(X,H)$ map shows a reversal in the magnetic contrast, which is attributed to a flip in the magnetization of the tip and is used to quantify its switching field (Figure S3c,d).

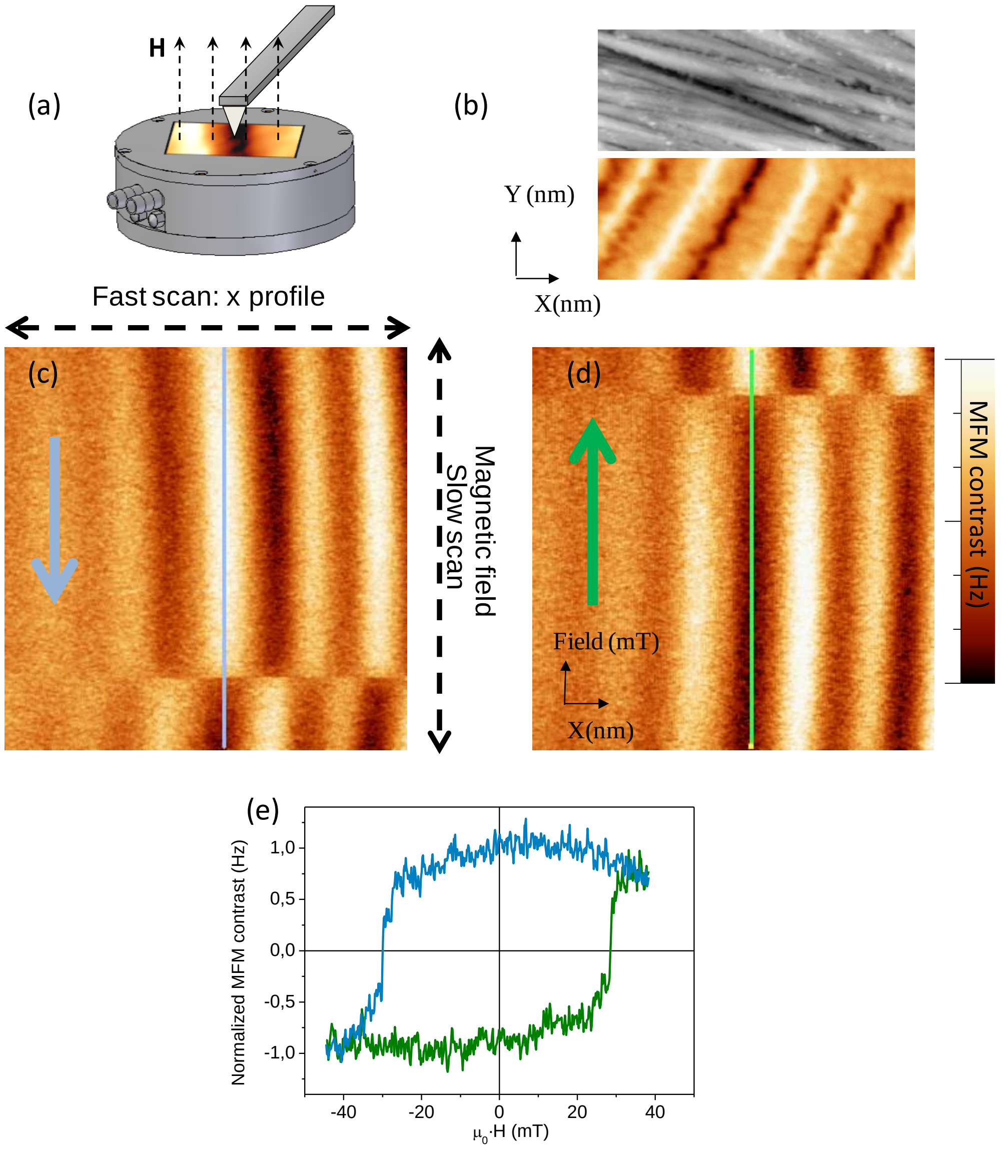

Figure S3: Switching field of MFM tips. (a) Applying OOP fields with an electromagnet, (b) topographic and MFM images are obtained. (c,d) Using the 3D modes, a contrast inversion reflects the switching field of the probe. (e) Evolution of the contrast in the 3D mode images.

Using this method, the switching field of different custom-made probes was studied and is shown in Figure S4. As can be seen there, there is a correlation with the in-plane coercive field of the corresponding reference thin film, measured by VSM.

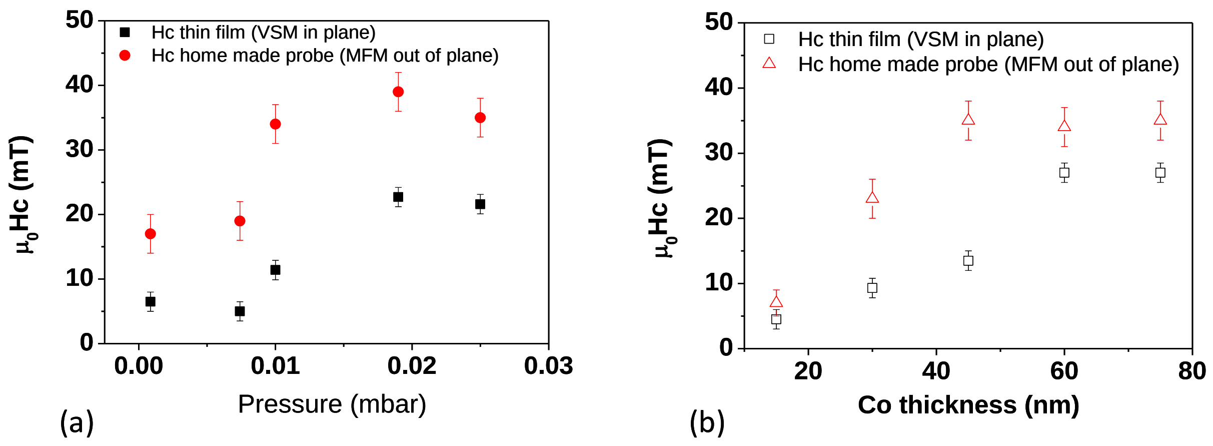

Figure S4: Evolution of the coercive field as a function of (a) the deposition pressure (for 45 nm thickness) and (b) Co layer thickness (for a deposition pressure of $1{\cdot}10^{-2}$ mbar).

3 Evaluation of the tip radius of the MFM probes

The tip radius was evaluated before and after deposition of the magnetic coating by imaging a carbon nanotube and using the following expression [3], where *w* and *h* are the apparent width and height of the nanotube:

$$R_{tip} = \frac{w^2}{8 \cdot h}$$

The results are presented in Figure S5, where an increase in the tip radius of only 0.5–1.0 nm is estimated for the case of a custom-coated probe with a 25 nm Co thickness on one of the pyramid sides. A much larger increase of a factor of about three with respect to their AFM counterparts is obtained for fully coated commercial probes.

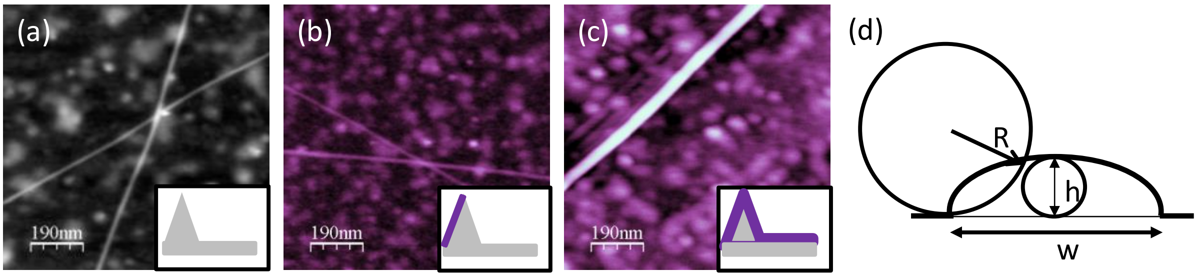

Figure S5: Calibrating the tip radius. Topography of carbon nanotubes measured with (a) standard AFM probe (Nanosensors PPP-FMR with no magnetic coating), (b) the same probe with a 25 nm thick Co coating on one side, (c) commercial MFM probe with a 50 nm thick CoCr coating and (d) sketch of the model used in [3], where R = tip radius, = nanotube height, w = apparent width of the nanotube.

4 Non-standard cantilevers for optimized MFM in liquid and vacuum environments

In scanning probe microscopy, the resonance frequency of the vibrating cantilever shifts, in the presence of a force gradient, inversely proportional to $\sqrt{k_0}$:

$$\omega = -\frac{\omega_0}{k} \cdot \frac{\partial F}{\partial z} = -\frac{1}{\sqrt{k_0 \cdot m}} \cdot \frac{\partial F}{\partial z}$$

Thus, choosing a cantilever with a smaller spring constant potentially enhances the sensitivity. However, this also reduces its natural resonance frequency, which results in a larger noise level, worsening the frequency shift detection. Such frequency dependent noise varies proportionally to $\sqrt{1/_{k \cdot Q}}$, with Q being the quality factor of the resonance. According to this, the measurement noise in liquid media is predicted to be four times larger using a standard cantilever with $k \approx 3$ N/m, compared to a typical one designed for this purpose (for instance, Olympus BL-AC40TS-C2) [4]. On the other hand, cantilevers with higher spring constants (20–40 N/m) are mostly used in vacuum environments. Despite them having a sensitivity about three times smaller than the standard ones, the fundamental noise level is improved by a factor of five, resulting in a improvement by a factor of ca. 1.4 in the frequency shift sensitivity (or, in other words, the MFM sensitivity). In addition, these hard levers yield a significantly larger mechanical stability that improves also the imaging quality.

This gives a ways to determine the most advantageous cantilever to optimize MFM imaging for each experiment. However, typical MFM probes currently available are mounted on cantilevers with resonance frequencies and spring constants of about 70 kHz and 1–3 N/m, which are most suitable for ambient pressure conditions. This presents an important limitation for optimized MFM in vacuum and liquid media.

For this reason, having the possibility to custom-coat tips in a quick and simple way permits to address this problem. Figure S6 presents two examples of this applicability for non-standard conditions: a hard cantilever –typically used in vacuum environments– with a resonance frequency and spring constant of around 300 kHz and 40 N/m, respectively, and a soft cantilever –for measurements in liquids– with a nominal resonance frequency and spring constant of 70 kHz and 0.09 N/m.

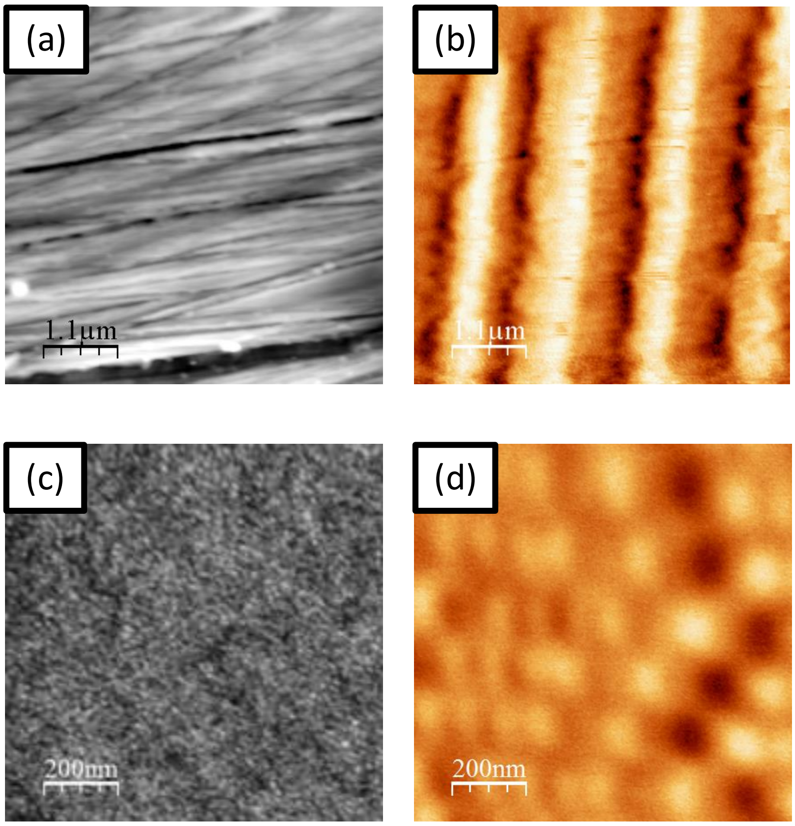

Figure S6: (a) Topography and (b) MFM images of a low-density hard disk acquired with a custom-made MFM probe mounted on a hard cantilever (40 N/m). Panels (c) and (d) show the analogous images of a high-density hard disk measured with a soft cantilever (0.09 N/m). Both probes were coated on one side with a 30 nm thick Co layer.

5 Compositional images acquired with the custom-coated MFM probes

In addition to its intrinsic high resolution capability, one of the main features of dynamic AFM is its ability to simultaneously detect different interactions. An example of compositional phase imaging using our custom-made probes is presented here, where an outstanding lateral resolution of 5 nm is achieved. According to the famous Cleveland's formula [5], if one assumes both the PLL and the topographic feedbacks to work ideally, the frequency shift measured during the topographic scan (Figure S7b,e) yields a map of the energy dissipation during the experiment. Notice that, during the retrace scan, no feedback is used to keep the oscillation amplitude constant so this assumption does not hold during MFM imaging. In general, different materials are expected to interact and dissipate energy in different ways; therefore, these images give an idea of the compositional distribution.

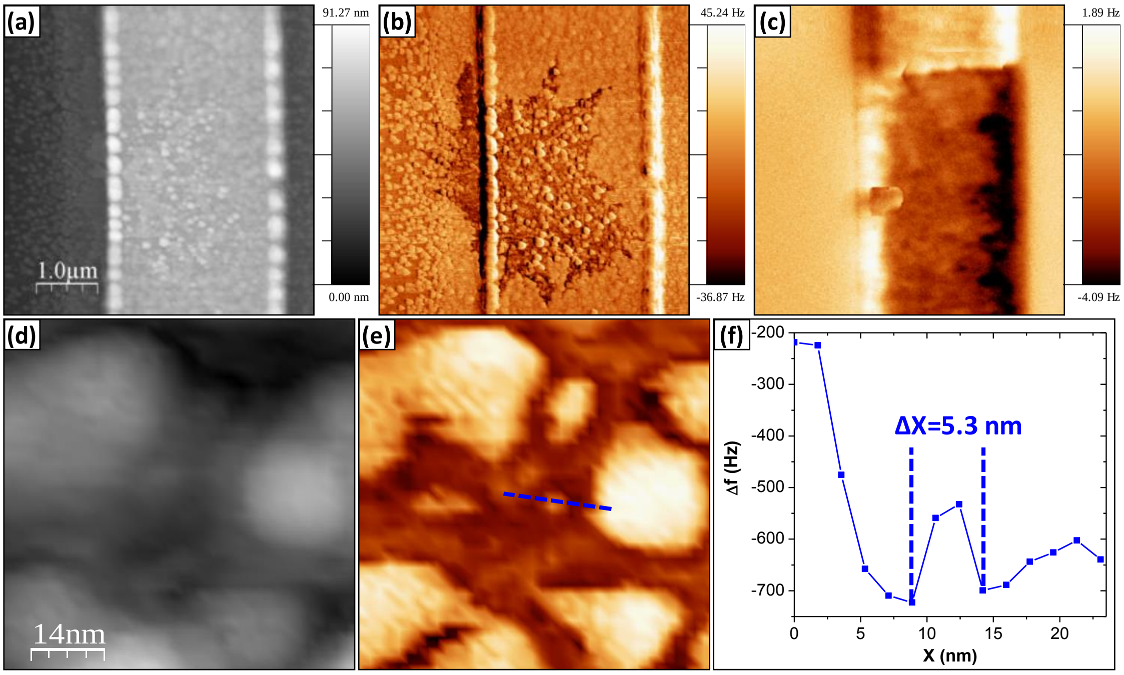

Figure 5: Compositional phase imaging. (a,d) Topography and (b,e) frequency shift maps obtained during the same scan. (c) Corresponding MFM image of the same region. (f) Profile along the dashed line marked in (e), showing a local frequency variation of 190 Hz and a spatial resolution of 5 nm.

Figure S7 presents an epitaxial Co(111) stripe lithographed onto vicinal Si(111) [6]. The frequency shift image (Figure S7b) recorded during the topographic scan (Figure S7a) reveals a well-defined dark stain covering a big part of the epitaxial Co nanostripes, whereas no remarkable feature is observed in the topographic image. Eventual electrostatic effects are unlikely to be responsible for it, as no relevant frequency shifts are recorded during the retrace scan in the MFM image (Figure S7c). In fact, this contrast can be associated to the adsorption of certain species in that region. A zoom-in image of the compositional map is displayed in Figure S7e, where a remarkable lateral resolution of 5 nm is achieved with a signal-to-noise ratio around 400. Notice that the corresponding magnified topography (Figure S7d) lacks such

outstanding detailed information; in fact, it is customary in scanning probe experiments to find better resolutions in the dissipation images.

References

[1] Phillips, G. N.; Siekman, M.; Abelmann, L.; Lodder, J. C. *Appl. Phys. Lett.* **2002,** *81,* 865-867. doi:10.1063/1.1497434

[2] Jaafar, M.; Serrano-Ramón, L.; Iglesias-Freire, O.; Fernández-Pacheco, A.; Ibarra, M. R.; De Teresa, J. M.; Asenjo, A. *Nanoscale Res. Lett.* **2011,** *6,* 407. doi:10.1186/1556-276X-6-407

[3] Wang, Y.; Chen, Y. *Ultramicroscopy* **2007,** *107,* 293. doi:10.1016/j.ultramic.2006.08.004

[4] Ares, P.; Jaafar, M.; Gil, A.; Gómez-Herrero, J.; Asenjo, A. *Small* **2015**, *11,* 4731–4736. doi:10.1002/smll.201500874

[5] Cleveland, J. P.; Anczykowski, B.; Schmid, A. E., Elings, V. B. *Appl. Phys. Lett.* **1998,** *72,* 2613. doi:10.1063/1.121434

[6] Ivanov, Yu. P.; Iglesias-Freire, O.; Pustovalov, E. V.; Chubykalo-Fesenko, O.; Asenjo, A. Phys. Rev. B 2013, 87, 184410. doi:10.1103/PhysRevB.87.184410
